# On Using Electric Circuit Models to Analyze Electric Field Distributions in Insulator-Based Electrokinetically Driven Microfluidic Devices

**DOI:** 10.3390/mi16111254

**Published:** 2025-11-01

**Authors:** J. Martin de los Santos-Ramirez, Ricardo Roberts, Vania G. Martinez-Gonzalez, Victor H. Perez-Gonzalez

**Affiliations:** School of Engineering and Sciences, Tecnologico de Monterrey, Av. Eugenio Garza Sada 2501, Monterrey 64700, NL, Mexico; martin.dlsr@tec.mx (J.M.d.l.S.-R.); ricardo.roberts@tec.mx (R.R.); a01411723@tec.mx (V.G.M.-G.)

**Keywords:** electrokinetics, FEM, electric field, microfluidics, resistance, circuits

## Abstract

Predicting the electric field distribution inside microfluidic devices featuring an embedded array of electrical insulating pillars is critical for applications that require the electrokinetic manipulation of particles (e.g., bacteria, exosomes, microalgae, etc.). Regularly, these predictions are obtained from finite element method (FEM)-based software. This approach is costly, time-consuming, and cannot effortlessly reveal the dependency between the electric field distribution and the microchannel design. An alternative approach consists of analytically solving Laplace’s equation subject to specific boundary conditions. This path, although precise, is limited by the availability of suitable coordinate systems and can only solve for the simplest case of a single pair of pillars and not for a rectangular array of pillars. Herein, we propose and test the hypothesis that the electric field across a longitudinal path within the microchannel can be estimated from an electric circuit model of the microfluidic device. We demonstrate that this approach allows estimating the electric field for whatever pillar shape and array size. Estimations of the electric field extracted from a commercial FEM-based software were used to validate the model. Moreover, the circuit model effortlessly illustrates the relationships between the electric field and the geometrical parameters that define the microchannel design.

## 1. Introduction

Electrokinetic (EK) phenomena (i.e., phenomena that produce motion of a fluid or a solid when in the presence of an electric field) have been extensively explored for the controlled manipulation of fluids and suspended particles within microfluidic devices [1,2,3,4]. EK methods exploit electroosmosis (EO), electrophoresis (EP), dielectrophoresis (DEP), electrorotation (ER), and other phenomena to either separate, concentrate, isolate, or characterize particles of interest (e.g., bacteria, viruses, parasites, nucleic acids, proteins, exosomes, and cells, among others) [5,6,7,8,9,10,11,12,13,14,15,16,17,18,19]. A first step towards this broad objective is the tailored design of the microfluidic device to achieve the needed manipulation of a given particle of interest. Specifically, the design of insulator-based electrokinetically driven (iEK) microfluidic devices—an alternative to microelectrode-based microfluidic devices [20,21] and optically induced DEP (also known as optoelectronic tweezers) [18,22,23], which uses insulating pillars to create a nonuniform spatial distribution of the electric field—must take into consideration many geometrical parameters, including, length, width, and height of the channel, size of the rectangular pillar array (rows and columns), separation between pillars in the array, and shape of the pillars (which may include its own list of defining geometrical parameters) [9,24,25,26,27,28,29,30,31,32].

Central to the effective design of iEK microfluidic devices is a thorough knowledge of the spatial distribution of the electric field throughout the channel. To obtain such knowledge, it is common practice to rely on software based on the finite element method (FEM) to solve Laplace’s equation subject to suitable boundary conditions to accurately describe the experimental conditions [8,9,33,34,35,36,37,38,39,40,41,42]. COMSOL Multiphysics and ANSYS Maxwell are two such commercially available FEM-based software packages. However, despite the accuracy of the electric field predictions produced with either software, time-consuming and computationally expensive parametric sweeps are needed to empirically unveil the relationship between the electric field distribution and the several geometrical parameters that define the iEK design [43,44]. Moreover, the acquisition cost of a teaching or research license of either software is high, potentially limiting the extent of the experimental efforts of a project by consuming a significant amount of the available funds.

A few efforts have been made to produce an analytical solution for the electric field spatial distribution within iEK devices. Cardenas-Benitez et al. used bipolar coordinates to produce a closed-form expression for the electric field across the central cutline of a DC-iEK microfluidic device [34]. The design of the modeled microfluidic device included only one pair of pillars, each with a circular footprint. However, as the literature demonstrates, a circular pillar footprint represents only one of many other possible footprints, including (but not limited to) elliptic, rhombic, rectangular, triangular, and even asymmetrical footprint designs [10,13,28,42,44,45]. Trying to circumvent this limitation, Ruz-Cuen et al. developed a model for triangular pillars with a rounded apex assuming hyperbolic patterns in an elliptic coordinate system [35]. Nonetheless, their model also considered only one pair of pillars. This is a hindrance as most iEK devices include a rectangular array of pillars [41,44,46].

In this contribution, we present an approach based on electric circuit theory to predict the spatial distribution of the electric field within iEK microchannels. Electric circuits have been used as tools in the design and characterization of iEK devices. For example, the Davalos research group exploited circuit theory to explain the coupling of the main microfluidic channel to the two lateral conductive channels present in their contactless DEP microfluidic devices and as a guide to define the thickness of their dielectric membranes [8]. Also, Perez-Gonzalez et al. used circuit theory to explain the increments in electric field and DEP force magnitudes at the gaps of DC-iEK devices, which were observed upon removal of columns from the pillar array [8,36]. Similarly, Zaman et al. adopted a circuit model to analyze optically induced dielectrophoresis systems [18]. Nonetheless, none of these contributions used circuit theory to produce estimations of electric field magnitude and distribution. Moreover, in the field of optical tweezers, electrical circuit equivalencies have been shown to explain when the system is activated and when it is not [19]. These equivalencies include capacitive structures with the aim of observing the AC response [18]. However, the method is based primarily on the conductivity and permittivity properties of the materials involved, as well as on dimensions, rather than on shape.

Other electrical techniques used in microfluidics that also rely on electric circuits to interpret data include resistive pulse sensing [47] and solid-state nanopores [48,49,50,51] wherein resistance estimates were generated for conical, cylindrical, hyperbolic, and bullet-shape single structures. Those estimates are based on the resistance equation proposed by Maxwell [52] and also aim to determine the electric field in those regions. However, they focus on very specific cases. At the same time, they highlight the need to use arbitrary geometries to estimate the effect of possible manufacturing defects.

Herein, we demonstrate that predicting the spatial distribution of the electric field within iEK microchannels is not only possible, but that the estimations follow the trends dictated by numerical models built in COMSOL Multiphysics (v6.0). Our electric-circuit-based method is a general one, that is, it can be used not only for designs featuring a single pair of pillars (of very specific footprint designs) like previous analytical efforts can [34,35], but for all pillar shapes and all rectangular array sizes. Moreover, because the model exploits the geometrical parameters of the pillars to produce equations that describe the electric field, the relationship between electric field magnitude and the microfluidic design becomes evident.

The remainder of this paper is organized as follows. Section 2 utilizes circuit theory to model iEK microfluidic devices. This model is firstly used to develop analytical solutions for a simple microfluidic channel constructed with a rectangular constriction at its midsection. This section ends by presenting a generalization for other types of constrictions. Section 3 focuses on describing the FEM-based model built for validating the electric circuit model. Section 4 compares the output of the models developed in Section 2 and software mentioned in Section 3. The evolving behavior of different types of constrictions represents the cornerstone of this analysis. Finally, Section 5 provides the conclusions of this work.

## 2. Model and Methods

### 2.1. Outline of the Problem

The objective of this research was to develop an electric circuit model of an electrically stimulated straight microfluidic channel which can predict the distribution of the electric field magnitude along a cutline parallel to the longitudinal axis of the channel. Assume that the longitudinal axis is oriented so that the inlet and outlet of the channel are located at its left and right ends, respectively. Also, the microchannel, made of an electrically insulating material with electrical conductivity $\sigma_{i}$, is filled with a liquid that exhibits electrical conductivity $\sigma \gg \sigma_{i}$, and stimulated with a voltage $V_{s}$. There are three regions in this microchannel: left, central, and right. The left and right regions are similar and characterized by total width $w_{t}$ and lengths $l_{l}$ and $l_{r}$, respectively. The central region has length $l_{c}$ and features a rectangular array of insulating pillars. The footprint of the pillars can take any form including rectangular, rhombical, circular, and elliptical, among others. Half-pillars, protruding from the two lateral walls of the central region of the microchannel, well-aligned with the rectangular array of pillars produce a rectangular array of a×b transversal gaps within the central region. The length of the microchannel is $l_{t}=\;l_{l}+l_{c}+l_{r}$ and its depth is D everywhere.

### 2.2. Building Block and Assumptions

Circuit theory assumptions include the lumped-element model, linearity, and instant information propagation. Easy-to-calculate parameters, such as voltage, current, and resistance, allow us to assess other variables, such as electric field distribution. The model to be described herein assumes a continuous medium and a thin electrical double layer (EDL) at the walls (so that surface charge will not cause an effect on the current because EDL dimensions are not comparable to the microchannel dimensions). Also, with this in consideration, no EDL overlaps are expected [53,54]. Therefore, the method models systems that are described by Laplace’s equation. It must be noted that, even in cases where a dilute solution is considered (e.g., deionized water), small trace amounts of ions are present, which can be estimated in the order of tens of µM via conductivity measurements. This, in turn, should return an EDL thickness in the order of ~100 nm, which, when compared to a typical microchannel depth with similar dimensions of ~30 µm as those considered here, represents less than 1% of the channel’s smallest dimension [38,55,56]. Therefore, the model remains valid considering the assumptions here described. However, in devices where the smallest dimension is closer to 1 µm, where the EDL thickness for dilute solitude represents a considerable portion of the device (i.e., PNP-NS scenarios), the model may be reconsidered.

Under these assumptions, consider any cuboid of length L, width W, and depth D made of a material with electrical conductivity σ. The electrical resistance across the length of this volume is

(1) $$R=\frac{L}{\sigma WD},$$

**Figure 1:**
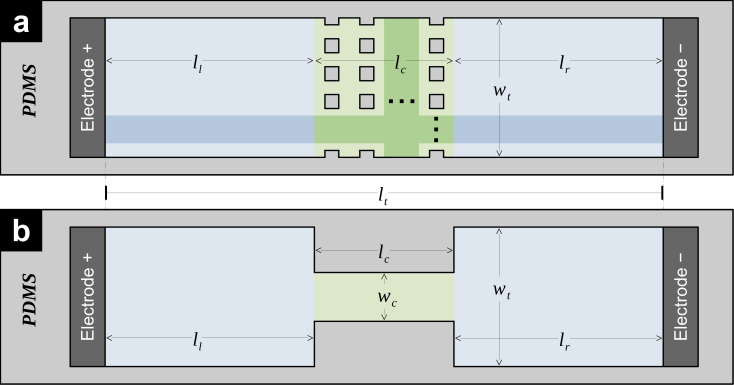
Schematic representations of (**a**) a microfluidic channel as described in the outline of the problem (square pillars are illustrated, however, the shape of the pillars can take any form); and (**b**) the simplest possible microfluidic channel containing only a pair of insulating pillars. A geometry similar to that shown in (**b**) will be used as a building block in the proposed model of a microfluidic channel like the one shown in (**a**).

Now, consider the simplest case of a microchannel with a singular rectangular gap in its central region (see Figure 1b). In this microchannel, a pair of insulating pillars (each featuring a rectangular footprint with length $l_{c}$ and width $\frac{w_{t}-w_{c}}{2}$) protrude from its lateral walls occupying its complete depth D. The left faces of the two pillars are located at a distance $l_{l}$ from the inlet of the microchannel. A length $l_{r}$ exists from the right faces of the two pillars to the outlet of the microchannel. Therefore, the length of the microchannel is $l_{t}=l_{l}+l_{c}+l_{r}$. This microchannel exhibits a width $w_{t}$ at the left and right regions, and a width $w_{c}$ in the central region. Also, the microchannel is filled with a liquid that has an electrical conductivity σ and is connected to a power supply $V_{s}$. It is clear that the liquid-filled microchannel can be represented by three cuboids, each of which exhibits an electrical resistance modeled by Equation (1). Then, assuming that the electric field magnitude changes abruptly at the left and right sides of the pillars, the electrical resistance of this microfluidic channel can be modeled as a series combination of three resistors, leading to the expression of total resistance of the microfluidic channel given by

(2) $$R_{T}=\frac{1}{\sigma D}\left(\frac{l_{c}}{w_{c}}+\frac{l_{l}+l_{r}}{w_{t}}\right)\;,$$

The following subsection demonstrates how, by using the assumptions presented herein, this three-section channel represents the basic building block necessary to construct the circuit model for a microfluidic channel comprising a rectangular array of pillars.

### 2.3. Electric Circuit Model of a Rectangular Array of Insulating Square Pillars Embedded in a Straight Microfluidic Channel

**Figure 2:**
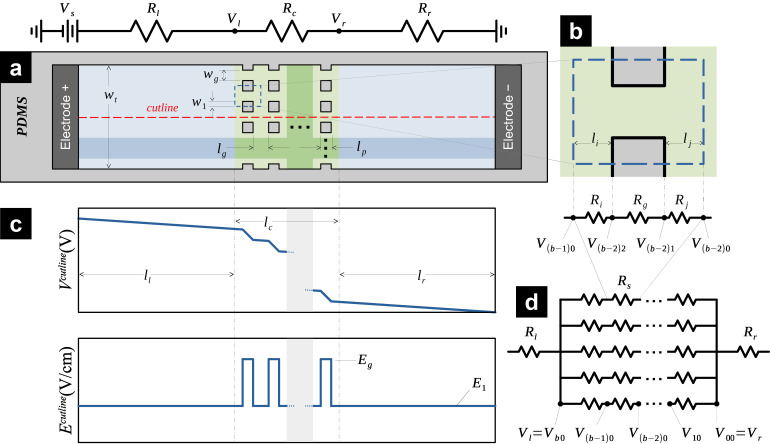
Voltage-drop and electric field intensity in a straight microfluidic channel with a rectangular array of electrically insulating square pillars. (**a**) Schematic of the microfluidic channel with an embedded array of square pillars; (**b**) single array element; (**c**) equivalent resistor circuit for a pillar array; and (**d**) electric potential and electric field distribution along the center cutline shown in (**a**).

We begin by adapting the general scenario described in the outline of the problem (Section 2.1), where the footprint of insulating pillars can take any shape, to the simpler case of a rectangular array of insulating square pillars (see Figure 2a). In this case, the left and right regions are unobstructed and have lengths $l_{l}$ and $l_{r}$, respectively. Also, both these regions have the same width $w_{t}$. Therefore, the resistance of the left and right regions are $R_{l}=\frac{l_{l}}{\sigma w_{t}D}$ and $R_{r}=\frac{l_{r}}{\sigma w_{t}D}$, respectively. The central region of length $l_{c}$ contains a rectangular array of insulating square pillars and two rows of insulating half-pillars located at the two longitudinal lateral walls. Each square pillar in the array has a side length $l_{p}$, while each pillar in the two rows of half-pillars has a length $l_{p}$ and width $w_{1}=\frac{l_{p}}{2}$. This arrangement of pillars creates a rectangular array of a×b transversal gaps, where a is the number of transversal gaps per column of the pillar array and b is the number of columns in the pillar array, respectively. The gap distance between pillar rows is $w_{g}=\frac{w_{t}-al_{p}}{a}$, while the gap distance between columns is $l_{g}=\frac{l_{c}-bl_{p}}{b}$. Note that $w_{g}=l_{g}$ when $w_{t}=l_{c}$ and a=b.

Fixing our attention on a sub-region surrounding and including any transversal gap in the pillar array (see the blue dashed rectangle in Figure 2a that is zoomed-in in Figure 2b), this sub-region resembles the single-gap case described in Section 2.2 (see Figure 1b) and it is clear that the whole pillar array can be constructed by joining several sub-regions transversally and longitudinally. Each of these sub-regions is comprised by a gap of width $w_{g}$ and a pillar length $l_{p}$, flanked by two pillar-free regions (i.e., three cuboids in total, all of them with conductivity σ, and each with a resistance that can be calculated using Equation (1)). Both of these pillar-free regions present the same width $\frac{w_{t}}{a}$ and lengths $l_{i}$ and $l_{j}$ (for the region to the left and the right of the cross-sectional gap, respectively). These lengths, when summed, yield the inter-gap distance $l_{g}$. Therefore, the resistance of a single inter-gap region is

(3a) $$R_{i}+R_{j}=\frac{l_{g}a}{\sigma w_{t}D}\;,$$

Conversely, the resistance of a single-gap region is

(3b) $$R_{g}=\frac{l_{p}}{\sigma w_{g}D}\;,$$

Since there are ab sub-regions in the pillar array, each formed by three cuboids, there are 3ab cuboids within the pillar array. Adding to this count are the left and right regions of the microfluidic channel; in total, there are 3ab+2 cuboids that can be thought of as electrical resistances. The resulting arrangement of resistances can be easily simplified using series and parallel equivalences commonly used in circuit theory. $R_{g}$ and $R_{i}+R_{j}$ denote the resistance of each individual gap and the inter-gap regions, respectively (see Figure 2d, Equations (3a) and (3b)). The electrical resistance of any given segment is denoted by

(4) $$R_{s}=R_{g}+R_{i}+R_{j}\;,$$

Therefore, the value of ${bR}_{s}$ corresponds to the resistance of a single row of transversal gaps. This value combined in parallel a times will yield the resistance value of the entire pillar array, denoted as $R_{c}$. In turn, $R_{l}$ and $R_{r}$ represent the remainder of the microfluidic channel to the left and right of the pillar array, respectively. The total resistance of the microfluidic channel is

(5) $$R_{T}=R_{l}+R_{c}+R_{r},$$

In this microfluidic channel, there are only two possible values of resistance per unit of length, which implies that there are only two possible magnitudes of electric field intensity, namely $E_{1}$ and $E_{g}$. As V=−∫Edl, the voltage drop across the channel will be greater in the regions where insulating pillars are present than in the regions where only conductive material fills the channel (see Figure 2c).

To calculate these two values of E, we start obtaining the voltage values of specific points at our microfluidic channel, namely $V_{l}$ and $V_{r}$ (see Figure 2d).

(6a) $$V_{l}=\frac{V_{s}\left(R_{c}+R_{r}\right)}{R_{l}+R_{c}+R_{r}}\;,$$

(6b) $$V_{r}=\frac{V_{s}R_{r}}{R_{l}+R_{c}+R_{r}},$$

Knowing $V_{s}$, $V_{l}$, $V_{r}$, and the fact that voltage falls at a constant slope at the left and right regions, we obtain the value of V as a function of x, where x is the distance advancing rightwards starting from the leftmost end (i.e., the inlet) of the microfluidic channel:

(7a) $$V(x)=V_{s}-\left(V_{s}-V_{l}\right)\left(\frac{x}{l_{l}}\right)\;,$$

for the region $0\leq x\leq l_{l}$ and

(7b) $$V(x)=V_{r}-V_{r}\left(\frac{x-l_{l}-l_{c}}{l_{r}}\right)\;,$$

for the region $l_{l}+l_{c}\leq x\leq l_{l}+l_{c}+l_{r}$. Note that Equations (7a) and (7b) denote simple interpolations. For both these regions, the magnitude of E is a simple derivative:

(8) $$E_{1}=-\frac{dV(x)}{dx}=\frac{V_{s}-V_{l}}{l_{l}}=\frac{V_{r}}{l_{r}},$$

To calculate E and V across the pillar array region, we use $V_{l}$ and $V_{r}$ (see Equations (6a) and (6b)) in the same way we used $V_{s}$ and the concept of ground in the previous step. In what follows, for a given row of gaps in the pillar array, voltages will have the structure $V_{nm}$, where 0≤n≤b and 0≤m≤2 are discrete indexes that describe any given gap position on the gaps row (see Figure 2b,d). For the n-th node, the resulting equation for $V_{n0}$ is as follows:

(9a) $$V_{n0}=V_{(n-1)0}+\frac{\left(V_{l}-V_{r}\right)}{b},$$

for n=[1,2,...,b]. Note that $V_{00}=V_{r}$ and $V_{b0}=V_{l}$. We use these voltage values to obtain the voltage at the beginning and end of each gap.

(9b) $$V_{n1}=V_{n0}+\frac{\left(V_{(n+1)0}-V_{n0}\right)\left(R_{j}\right)}{R_{g}+R_{i}+R_{j}},$$

(9c) $$V_{n2}=V_{n1}+\frac{\left(V_{(n+1)0}-V_{n1}\right)\left(R_{j}+R_{g}\right)}{R_{g}+R_{i}+R_{j}}\;,$$

for n=[1,2,...,b−1]. In total, the above equations solve for 3b+1 values of voltage across the microfluidic channel. These values and linear interpolations between them (see Equations (7a) and (7b)) fully describe the voltage at all points of a cutline parallel to the longitudinal axis of the channel passing exactly in between two rows of pillars (see Figure 2a).

The electric field at the inter-gap regions (such as the one indicated by $l_{g}$ in Figure 2a) is described by Equation (8). That leaves only the electric field at the gaps to be described, exploiting Equation (9a–c).

(10) $$E_{g}=\frac{V_{n2}-V_{n1}}{l_{p}},$$

for n=[0,1,...,b−1]. Note that, for this rectangular array of square pillars, $E_{g}$ yields the same result for any gap (i.e., any value of n).

Finally, the electric field amplification Ψ can be obtained with either the calculated electric fields of Equations (8) and (10) or the channel dimensional parameters, which yield

(11) $$\Psi =\frac{E_{g}}{E_{1}}=\frac{w_{t}}{aw_{g}},$$

In the following subsection, the equations for $R_{l}$, $R_{r}$, $R_{i}$, and $R_{j}$ will remain unchanged, and the method to obtain $R_{g}$ will be generalized.

### 2.4. Electric Circuit Model of a Rectangular Array of Insulating Arbitrary-Shape Pillars Embedded in a Straight Microfluidic Channel

**Figure 3:**
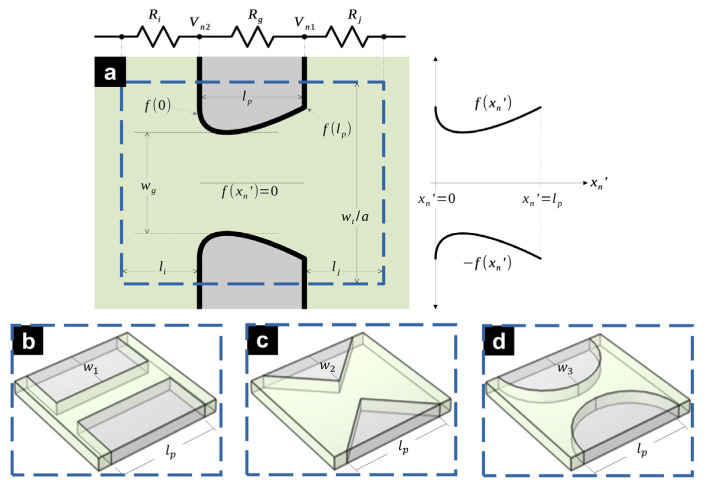
Relevant dimensional parameters of four different pillar designs. Top-view of (**a**) arbitrary pillar shape, and isometric view of (**b**) rectangular, (**c**) triangular, and (**d**) circular.

Herein, we use an arbitrary function $f\left({x_{n}}^{\prime }\right)$ and its negative $-f\left({x_{n}}^{\prime }\right)$ in the domain of $0\leq {x_{n}}^{\prime }\leq l_{p}$ to define our pillar walls (see Figure 3), which replaces $l_{p}$ and the square pillars it used to describe. Moreover, ${x_{n}}^{\prime }=x-l_{l}-\frac{\left(b-n-1\right)l_{c}}{b}-l_{i}$ for the domain n=[0,1,…,b−1]. For $f\left({x_{n}}^{\prime }\right)$ to fully describe an enclosed insulating pillar, the function needs to fulfill the condition $f\left(0\right)=f\left(l_{p}\right)=\frac{w_{t}}{2a}$. This condition allows single array elements of a given column to interconnect at ${x_{n}}^{\prime }=0$ and ${x_{n}}^{\prime }=l_{p}$ (see Figure 3c,d). This condition may not be true in some cases where it is necessary to add vertical walls that enclose the insulating region (see Figure 3a,b), which do not affect any calculation.

The greater magnitude of $f\left({x_{n}}^{\prime }\right)$ corresponds to a removal of insulating material. In contrast, $f\left({x_{n}}^{\prime }\right)=0$ represents the absence of a gap (i.e., a microchannel fully obstructed with insulating material). Therefore, the condition $f\left({x_{n}}^{\prime }\right)>0$ must be true for any working microfluidic channel. Conversely, $f\left({x_{n}}^{\prime }\right)=\frac{w_{t}}{2a}$ would represent complete absence of insulating material in the gap region (i.e., a channel void of insulating pillars). Therefore, the condition $0<f\left({x_{n}}^{\prime }\right)<\frac{w_{t}}{2a}$ is necessary for a pillar to exist throughout the pillar length, which is defined as $l_{p}$. Consequently, the generalized equation of $R_{g}$ is as follows.

(12) $$R_{g}=\frac{1}{2\sigma D}\int_{0}^{l_{p}}\frac{d{x_{n}}^{\prime }}{f\left({x_{n}}^{\prime }\right)},$$

while the method to obtain $R_{g}$ has changed, Equation (4) remains unmodified. Similarly, the gap between pillar columns of arbitrary shape remains as $l_{g}=\frac{l_{c}-{bl}_{p}}{b}$ while the gap between pillar rows is now defined as $w_{g}=min\left\{2f\left({x_{n}}^{\prime }\right)\right\}$. Having $w_{g}$ allows us to calculate $\Psi =\frac{w_{t}}{aw_{g}}$. With this, we conclude the general description of the proposed method.

It must be noted that, although iEK devices rarely include an array of arbitrarily shaped pillars, in this contribution we only analyzed a subset of the many different pillar shapes reported in the literature, leaving several designs out from our discussion (e.g., funnels [57] or asymmetrical pillars [58]). Moreover, our method can come in handy to describe fabricated geometries and not only ideal designed geometries. This is because even when a pillar is designed to have sharp tips (e.g., triangular), fabricated devices most commonly present rounded tips that can be accurately described by a parabolic or hyperbolic function, both of which could be described by our model. The following subsection will focus on obtaining specific solutions of $R_{g}$ as defined in Equation (12) for commonly used pillar contours.

### 2.5. Analytical Solution of Frequently Used Pillar Shapes

The method presented here slices the microfluidic channel into several segments. Most of these segments are cuboids, and therefore their electrical behavior can be easily calculated using Equation (1). The gap region, however, may present complex shapes. Therefore, our method now focuses on analytical expressions for $R_{g}$ in specific cases. While the method is able to solve for any value of $f\left({x_{n}}^{\prime }\right)$, we focus on four functions used to describe pillar footprints which are commonly used in insulator-based electrokinetic (iEK) microfluidics, namely, rectangular, triangular, circular, and elliptic [13,25,37,44,59,60,61]. For each shape, the same process is applied. Firstly, we invoke relevant antiderivatives and definite integrals that represent a foundation for the following steps, which were validated using WolframAlpha (see Table S1, https://www.wolframalpha.com/). Just as we defined $0\leq {x_{n}}^{\prime }\leq l_{p}$, we also set $-\frac{l_{p}}{2}\leq {x_{n}}^{\prime \prime }\leq \frac{l_{p}}{2}$ to simplify some selected equation solutions. Therefore, we define $f\left({x_{n}}^{\prime \prime }\right)$ where ${x_{n}}^{\prime \prime }={x_{n}}^{\prime }-\frac{l_{p}}{2}$. The resulting equations are presented in Table 1.

To obtain the voltage value at any given point ${x_{n}}^{\prime }$ within the gap, we use the obtained values of $R_{g}$ (see Table S2) and $R\left({x_{n}}^{\prime }\right)$ (see Table S3). Therefore, we obtain

(13) $$V\left({x_{n}}^{\prime }\right)=V_{n2}-\left(V_{n2}-V_{n1}\right)\left[\frac{R\left({x_{n}}^{\prime }\right)}{R_{g}}\right],$$

where $R\left({x_{n}}^{\prime }\right)$ represents the integration of a segment of arbitrary length within the gap region. Table 2 presents the results for the relevant cases. Once the voltage is calculated with Equation (13), the electric field for the same region is readily obtained.

(14) $$E\left({x_{n}}^{\prime }\right)=-\frac{dV\left({x_{n}}^{\prime }\right)}{d{x_{n}}^{\prime }}=\left(\frac{V_{n2}-V_{n1}}{R_{g}}\right)\left[\frac{dR\left({x_{n}}^{\prime }\right)}{d{x_{n}}^{\prime }}\right],$$

As the resistance is the integral of the gap function, the derivative of the resistance takes us back to the original function of resistivity, while the rest of the parameters are constants already obtained. Therefore,

(15) $$\frac{dR\left({x_{n}}^{\prime }\right)}{d{x_{n}}^{\prime }}=\frac{1}{2\sigma Df\left({x_{n}}^{\prime }\right)},$$

The resulting equations of electric field across commonly used gaps are presented in Table 2.

**Table 2:** Electric field as a function of position $f\left({x_{n}}^{\prime }\right)$ or $f\left({x_{n}}^{\prime \prime }\right)$ for commonly used pillar shapes.

Pillar Shape	$E\left({x_{n}}^{\prime }\right)$ $\mathrm{or}\;E\left({x_{n}}^{\prime \prime }\right)$
Rectangle	$\left(\frac{V_{n2}-V_{n1}}{R_{g}}\right)\left[\frac{1}{2\sigma D\left(\frac{w_{t}}{2a}-w_{1}\right)}\right]$ $\left(\frac{V_{n2}-V_{n1}}{R_{g}}\right)\left[\frac{1}{2\sigma D\left(\frac{w_{t}}{2a}-\frac{2w_{2}{x_{n}}^{\prime }}{l_{p}}\right)}\right]$ Simplifying $\left(V_{n2}-V_{n1}\right)\left[\frac{al_{p}}{R_{g}\sigma D\left(w_{t}l_{p}-4aw_{2}{x_{n}}^{\prime }\right)}\right]$
Triangle	$\left(\frac{V_{n2}-V_{n1}}{R_{g}}\right)\left[\frac{1}{2\sigma D\left(\frac{w_{t}}{2a}-2w_{2}+\frac{2w_{2}{x_{n}}^{\prime }}{l_{p}}\right)}\right]$ Simplifying, $\left(V_{n2}-V_{n1}\right)\left[\frac{al_{p}}{R_{g}\sigma D\left(w_{t}l_{p}-4al_{p}w_{2}+4{aw}_{2}{x_{n}}^{\prime }\right)}\right]$
Circle	$\left(\frac{V_{n2}-V_{n1}}{R_{g}}\right)\left\{\frac{1}{2\sigma D\left[\frac{w_{t}}{2a}-\sqrt{{\left(\frac{l_{p}}{2}\right)}^{2}-{\left({x_{n}}^{\prime \prime }\right)}^{2}}\right]}\right\}$
Ellipse	$\left(\frac{V_{n2}-V_{n1}}{R_{g}}\right)\left\{\frac{1}{2\sigma D\left[\frac{w_{t}}{2a}-\sqrt{{\left(w_{3}\right)}^{2}-{\left(\frac{2w_{3}}{l_{p}}\right)}^{2}{\left({x_{n}}^{\prime \prime }\right)}^{2}}\right]}\right\}$

While the resulting equations prove unwieldy, the resulting values work for either a single constriction or a pillar array. Therefore, we have solved for the voltage (as defined in Equation (13)) and electric field (as defined in Table 2) along a cutline that passes through the center of the gap between any two rows of pillars across the microfluidic channel. The following section will shortly digress into FEM-based simulation software and its limitations, while Section 4 will compare said software results with the model developed here.

## 3. FEM-Based Model

In order to test the accuracy of the circuit model, a set of relevant cases were analyzed with it and validated against COMSOL (v6.0)-based simulations (COMSOL Inc., Burlington, MA, USA). For the COMSOL model, the electric currents interface from the AC/DC module was used to solve Laplace’s equation. Each model relies on 2D geometries and stationary studies. Channel dimensions were established as 1 cm, 1 mm, and 20 μm for channel length $\left(l_{t}\right)$, width $\left(w_{t}\right)$, and depth $\left(D\right)$, respectively. Pillars shapes and dimensions were established as listed in either Section 4.1 or Table S4 (as needed). Figure S1 shows the domain and boundary conditions used for the electric currents interface. The blue domain corresponds to a liquid, while gray regions correspond to PDMS (the selected insulating material from which pillars are assumed to be fabricated). The electrical conductivity and relative permittivity of the liquid were set to values of 100 µS/cm and 80, respectively [42,43,62]. Moreover, the electrical conductivity of PDMS was set to 2.5 × 10-10 µS/cm [63,64] and its relative permittivity of 2.75 was defined by COMSOL’s material library. The mesh presents a free triangular distribution and implements two predefined domain types: Coarser quality for the major part of the microchannel and pillar array, and extremely fine element size for a virtual domain. The virtual domain (refined mesh-box) is localized along the center cutline (see Figure 2a), taking into account the near regions, to improve quality for FEM-calculations. Virtual domain also invokes a regular refinement three times.

## 4. Results and Discussion

### 4.1. Circuit Model Validation Against COMSOL Simulations

Numerical evaluations of the circuit model for rectangular, elliptical, and triangular pillars are presented in this subsection. Each design consists of seven rows (*a* = 7) and one column (*b* = 1) with $w_{t}=1$ mm, D=20 μm, $w_{1}=w_{2}=w_{3}=375$ μm, and $l_{p}=l_{c}$. Specifically, to model rectangular and triangular pillars, $l_{c}=800\;$μm and $l_{l}=l_{r}=4.6$ mm. Meanwhile, to model elliptical pillars, $l_{c}=750$ μm and $l_{l}=l_{r}=4.625$ mm. All models generate an electric field by applying 100 V across the channel electrodes (see Figure 1 and Figure 2a). These particular values of voltage and dimensional characteristic are selected as they are representative of the values found in published works [31,35,38,61]. Predictions from the circuit model were obtained from MATLAB R2025a (MATHWORKS, Natick, MA, USA).

**Figure 4:**
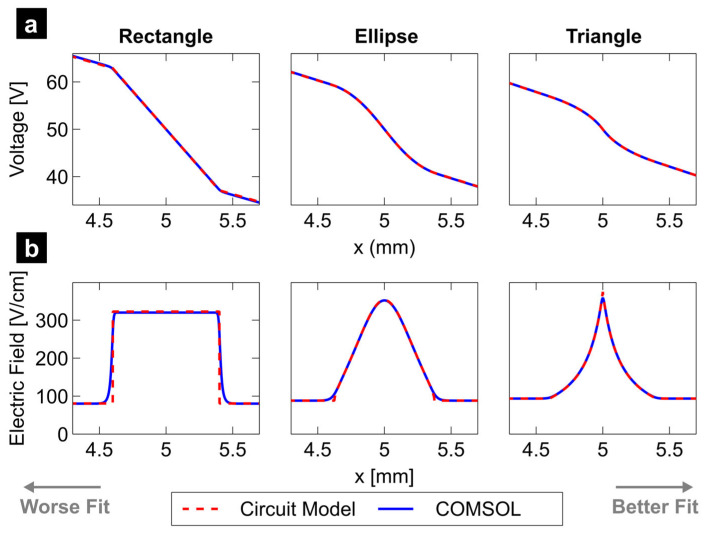
Electrical behavior of rectangular, ellipsoidal, and triangular pillar arrays. Comparison between the proposed circuit model and COMSOL simulations for voltage (**a**) and electric field (**b**).

Figure 4a,b show voltage and electric field distributions along the center cutline for the three pillar shapes. For two out of the three pillar shapes, the circuit model (red dashed line) predicts more abrupt changes in the electric field than the COMSOL simulation (blue solid line), which presents smoother curves. While the maximum electric field is almost identical for all cases, it is the triangular pillar geometry that presents the most similarity between the predictions obtained from the circuit model and COMSOL simulation. For the case of the ellipse-shaped pillars, both the circuit and COMSOL models predict similar profiles, except at the entrance to the interpillar-gap region, where a visible deviation occurs. The rectangular pillars present a visible deviation between the two predictions in the same region. This was expected given the strong geometry dependence of the circuit model on the pillar geometry.

Because the model assumes perfect resistors that exhibit a uniform electric field magnitude within, when the device design under analysis includes insulating walls perpendicular to the trajectory of the otherwise uniform electric field, the model predicts a discrete (rather than continuous) change in the magnitude of the field. Pillars with rectangular shapes exhibit exactly this behavior (see Figure 4b—left). At the other end of the spectrum, triangular shapes, when finely discretized, can easily approximate the continuous gentle shift in electric field magnitude predicted by COMSOL simulations (see Figure 4b—right). The elliptic-shaped pillars represent a midpoint (see Figure 4b—center).

### 4.2. Equivalencies Between Arrays of Insulating Pillars

**Figure 5:**
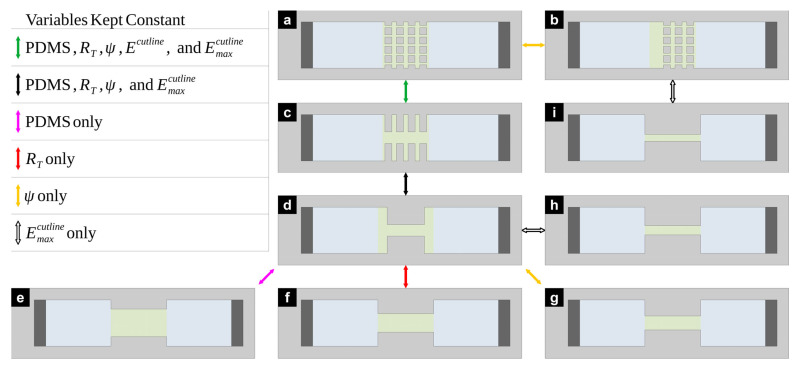
Microfluidic channel design variations that maintain selected parameters or channel behavior. Namely, the amount of PDMS volume used by channel structures, electrical resistance $R_{T}$, electric field throughout the cutline $E^{cutline}$, electric field amplification factor Ψ, and maximum magnitude of the electric field $E_{max}^{cutline}$. (**a**) (5x4) pillar array; (**b**) (5 × 3) array with pillars having same dimensions as those in (**a**); (**c**) (1 × 4) array, an equivalence of (**a**); (**d**) (1 × 1) array, an equivalence of (**c**); (**e**) (1 × 1) array with very large *w_g_*; (**f**) (1 × 1) array with large *w_g_*; (**g**) (1 × 1) array with smaller *w_g_* than (**d**); (**h**) (1 × 1) array with very small *w_g_*; (**i**) 1 × 1) array with the smallest *w_g_* in this figure, and with more PDMS than (**b**). Note that (**e**), (**f**), (**g**), and (**h**) derive from (**d**).

While the set of pillars presented in Section 4.1 comprise multiple base designs, the sets of pillars selected for this section focused solely on rectangular structures as these facilitated the proposed analysis. Variations in the parameters—pillar width $\left({2w}_{1}\right)$, length $\left(l_{p}\right)$, and the number of rows $\left(a\right)$ and columns $\left(b\right)$—were tested. The following results show that a particular set of changes in a channel can maintain a relevant parameter constant. A broad set of such changes can be found in Figure 5.

For any given microfluidic channel design, it is possible to propose an alternative design that maintains one or more relevant parameters. The more parameters one aims to maintain, the narrower the possibility space. The parameters used in the following analysis are the PDMS volume used by pillars and constrictions on the channel, electrical resistance $R_{T}$, electric field amplification factor Ψ, and maximum magnitude of the electric field across the cutline $E_{max}^{cutline}$. It is possible to transform a pillar array (Figure 5a) into a set of constrictions (Figure 5c) and maintain all these parameters (Figure 5 green arrow). Not all transformations will produce the same effect. Case in point, a transformation of the said series of constrictions (Figure 5c) into a single constriction (Figure 5d) will inevitably show a different $E^{cutline}$, while the rest of the parameters remain constant (Figure 5 black arrow). This symmetry is only possible when the material is kept constant and displaced only on its x axis. More elaborate changes in the PDMS structures will most likely affect all parameters. Maintaining one parameter is possible with the correct selection of dimensional parameters. Note, however, that the transformation that maintains the amount of PDMS (Figure 5 pink arrow) will be different to the transformation that keeps $R_{T}$ constant (Figure 5 red arrow). The same is true for transformations that maintain Ψ (Figure 5 yellow arrow) and $E_{max}^{cutline}$ (Figure 5 white arrow).

It is not necessary to invoke circuit theory to identify a microfluidic channel pair with the same amount of PDMS (Figure 5 pink arrow), it is only necessary to compensate between added and removed material. For the particular case of rectangular pillars, the multiplication $l_{p}w_{1}$ must remain constant. Figure 5d,e fulfill this condition, ${PDMS}_{D}={PDMS}_{E}$. In contrast, one does need circuit theory to pick out two microfluidic channels that share the same electrical resistance (Figure 5 red arrow). For this procedure, it is necessary to calculate the resistance of each microfluidic system (*R_T_*, see Equation (5)), equate them, and solve for the desired restrictions. For the particular case of rectangular pillars, one can set a new channel width and find the corresponding channel length, or the other way around. Figure 5d,f fulfill this condition of $R_{D}=R_{F}$. Just like the case that maintains PDMS, a purely dimensional approach is sufficient to find two microfluidic channels that preserve their amplification factor (Figure 5 yellow arrow). Namely, the ratio between the region of largest and smallest aperture must be found for each case and equated. For the particular case of rectangular pillars, the gap width $w_{g}$ must be kept constant while the pillar length $l_{p}$ can be changed arbitrarily. Figure 5a,b $\left(\Psi_{A}=\Psi_{B}\right)$, and Figure 5d,g $\left(\Psi_{D}=\Psi_{G}\right)$ fulfill this condition. Finally, to obtain two microfluidic channels that present the same maximum electric field (Figure 5 white arrow), one must invoke both circuit theory and dimensional parameters of the channel. Namely, it is necessary to obtain the channel maximum magnitude of the current density:

(16) $$J_{max}=\frac{V_{s}}{{Dw}_{g}R_{T}},$$

Note that the parameter D cancels out when comparing two channels with equal $J_{max}$ and depth to solve for the desired restrictions. Equation (16) works for any pillar shape. Figure 5b,i $\left(E_{maxB}=E_{maxI}\right)$, and Figure 5d,h $\left(E_{maxD}=E_{maxH}\right)$ fulfill this condition.

**Figure 6:**
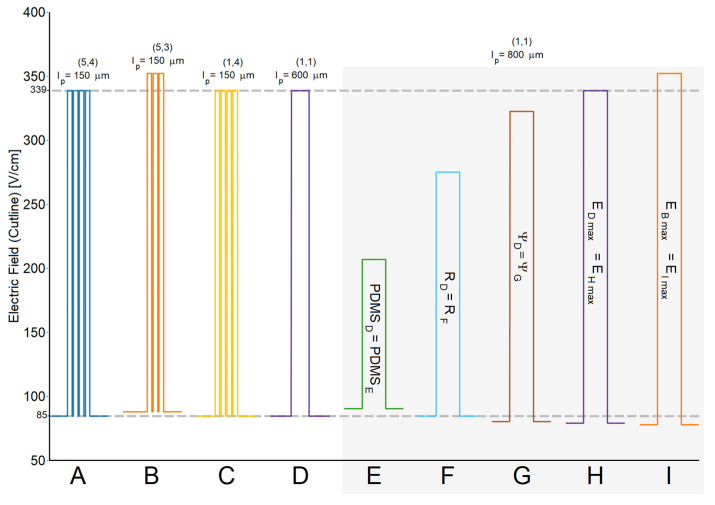
Calculated electric field profiles for selected microfluidic channel cases. Cases E, F, G, and H share one distinct parameter of case D each. Namely, the same amount of PDMS, resistance, amplification factor, and maximum electric field, respectively. Cases B and I share the same value of maximum electric field. Numbers within parentheses represent (*a*, *b*).

Previously, in Section 4.1, we validated the circuit model of a single gap against COMSOL simulations (see Figure 4). To validate the accuracy of the circuit model through the central cutline of all microchannels illustrated in Figure 5, the dimensional parameters $a,b,w_{1},l_{p}$, and $w_{g}$ listed in Table S4 were used to evaluate Equations (5), (11) and (14). Moreover, the results obtained from the circuit model were validated against results obtained from the COMSOL model described previously in Section 3. Results are also listed in Table S4. Furthermore, Figure 6 shows the detailed distortion of the electric field as predicted by the circuit model across a section of a cutline similar to that shown in Figure 2a. Specifically, the graph focuses on the pillar region of the different channels (i.e., a 2 mm long segment at the center of the cutline). Two gray horizontal lines indicate the maximum and minimum magnitudes of the electric field of case A as a reference. Note that the cases under gray shade correspond to a set of channels with a single rectangular constriction and constant pillar length (*l_p_* = 800 μm) with progressively more PDMS for each case, as explained in the previous paragraphs. Array size and pillar length is recorded at the top of each case. Key equivalences from Figure 5 are also included.

Confirming the equivalences illustrated in Figure 5, Figure 6 demonstrates that cases A, C, D, and H reach the same value of $E_{max}$ despite presenting different array and gap configurations. The same is true for the B and I case pair. When $V_{s}$ and D have a unique value for several microchannel designs, all channels will achieve the same $E_{max}$ if the value $w_{g}R_{T}$ is the same for all. This occurs because current density remains the same as in Equation (16). Note that the case pair D and F share both electrical resistance and $E_{min}$, which in turn points to the same value of current flowing across both channels. Figure 6 also exhibits deformations of the electric field caused by PDMS structures.

### 4.3. Discussion

**Figure 7:**
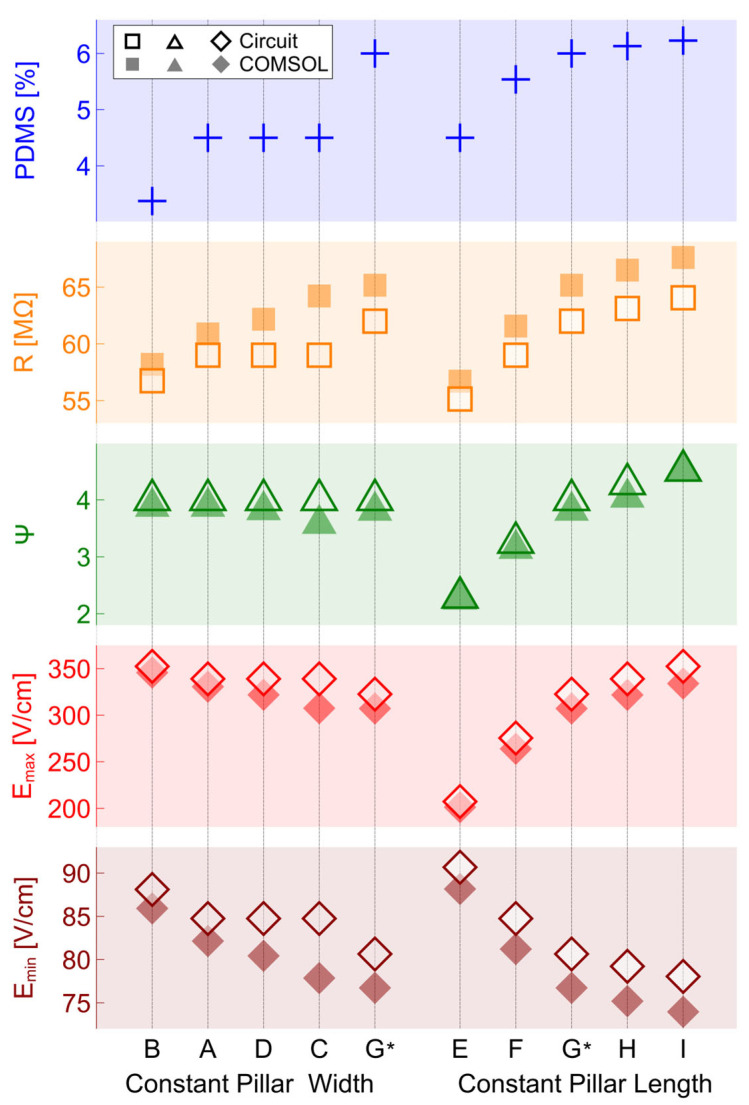
Selected cases grouped on two different patterns of PDMS addition and their corresponding behavior. Data for both the circuit model and corresponding COMSOL simulations. Cases B, A, D, C, and G show incremental amounts of PDMS added horizontally (constant total gap width $aw_{g}$). Cases E, F, G, H, and I show incremental amounts of PDMS added vertically (constant pillar length). * Note that G is present in both patterns.

Through the electric field magnitude plots, Figure 6 indirectly gave an idea of other relevant parameters such as the channel electrical resistance or amplification factor. Figure 7, in turn, explicitly shows these values (obtained from the circuit model and from COMSOL). Moreover, it presents the cases ordered by abundance of PDMS (i.e., an electrically insulating material) in the channel and the location in which said material is added by showing two progression patterns. This was decided based on the many different pillar arrays used in iEK microfluidic designs available in the literature that, while exploring different pillar shapes and array sizes, implicitly impact the amount of insulating material present in their fabricated microfluidic channels. The leftmost grouping of cases (B, A, D, C, G) presents constant pillar width $w_{1}$ and adds PDMS by increasing pillar length $l_{p}$. Conversely, the rightmost grouping of cases (E, F, G, H, I) presents constant pillar length and adds PDMS by increasing pillar width. Note that case G is a shared point in both case groupings. Figure 7 also presents values of resistance, amplification factor, maximum electric field and minimum electric field for both the circuit model proposed in this paper and COMSOL simulations. Finally, note that cases D and G present the same amplification factor, while the rest of the parameters differ.

Figure 7 shows a positive correlation between PDMS addition and electrical resistance for both of the case groupings presented. Conversely, it also shows a negative correlation between PDMS and $E_{min}$ for both case groupings. The amplification factor shows a more nuanced behavior, where the circuit model shows positive correlation to PDMS only when pillar length is maintained constant. Finally, $E_{max}$ presents the most divergent behavior. Namely, it presents a negative correlation when PDMS is added while maintaining pillar width and a positive correlation when PDMS is added while maintaining pillar length. It is noteworthy that, for the circuit model, the geometrical differences between cases A, C, and D produce no alteration in the electrical response of the corresponding microchannels. In contrast, the COMSOL model exhibits that the electrical resistances of these three cases are, in fact, different, therefore affecting the electrical response of the corresponding microchannels. This is a limitation imposed by the assumption of abrupt change in the electric field magnitude at the left and right sides of the pillars, which is required to construct the circuit model described herein.

**Figure 8:**
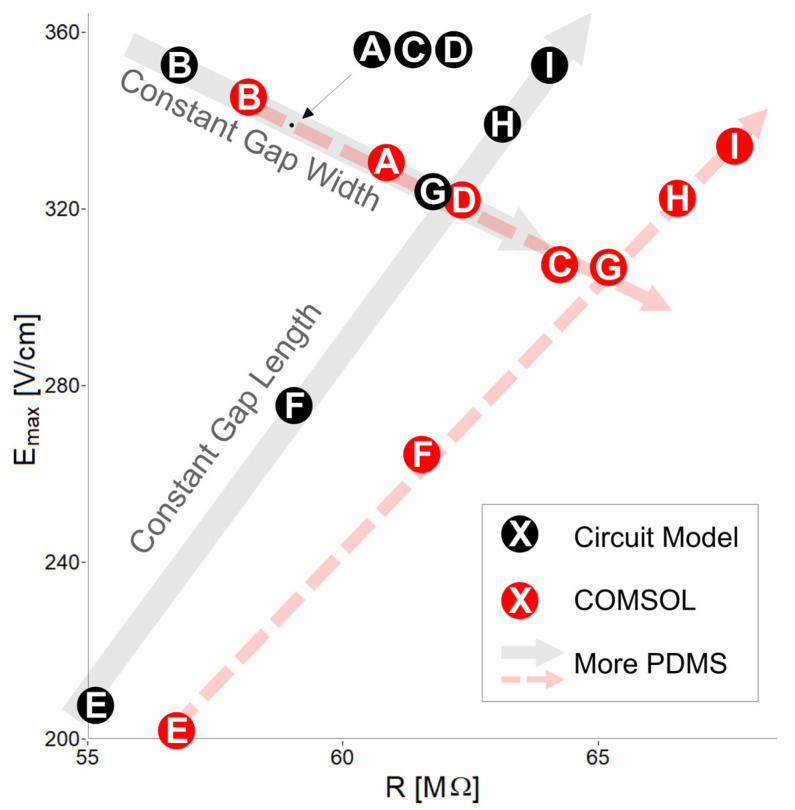
Paths of pure horizontal and vertical PDMS addition for the circuit model and COMSOL simulation. Gray and red arrows indicate general trends of addition of PDMS. Black and red points denote circuit model and COMSOL results, respectively. Keep in mind that, regardless of their separation along one red arrow, cases A, C, and D have the exact same amount of PDMS.

These variabilities of correlations beg for further analysis. Specifically, in cases where both the addition or removal of PDMS can produce the same outcome (when performed correctly), it is important to know each option’s second-order effects. Figure 8 allows us to see these two paths in a single chart for $E_{max}$ and resistance. It is possible to see in cases B and I that both the addition and removal of PDMS increase $E_{max}$. Either change, however, moves the electrical resistance of the channel to very different values. The circuit model and COMSOL simulations agree on this point.

Adding more PDMS longitudinally while keeping $aw_{g}$ constant in cases B, A, C, D, and G increases resistance (because there is more insulating material). Also, adding more rows in the pillar array while keeping $aw_{g}$ constant creates several bottlenecks for the electric field, reducing its magnitude. Adding PDMS transversally to reduce the gap width, as in cases E, F, G, H, and I, results in increased resistance because there is more insulating material; however, the maximum electric field increases. This occurs because the electric field lines are forced to pass through a single narrower constriction. As seen in electrical circuits, most of the current will always seek the path of least resistance. Placing several constrictions perpendicular to the electric field direction (i.e., several pillar rows) reduces the current traveling between them, thereby reducing the maximum electric field.

### 4.4. Application of the Model

In order to assess the applicability of the model in a practical scenario, we evaluated the designs proposed by Gallo-Villanueva et al. where Joule heating was evaluated for six different DC-iEK microfluidic designs [43]. In such cases, temperature rise is dependent on the electric field magnitude (influencing the heat source). Therefore, herein, the scope will be limited to modeling the behavior of the electric field.

The analyzed devices feature rectangular arrays of two pillar shapes, namely elliptic and rhombic, with three design variations for each geometry designated as original, suboptimized, and optimized [43]. For simplicity, in this study, the original, suboptimized, and optimized adjectives were replaced by labels D1, D2, and D3, respectively. Using the equivalencies shown in Section 4.2, all designs were converted to single constriction designs. A summary of all design parameters and equivalencies is presented in Table 3. Note that, when converted to a single constriction configuration, a rhombic array is reduced to a triangular geometry.

**Figure 9:**
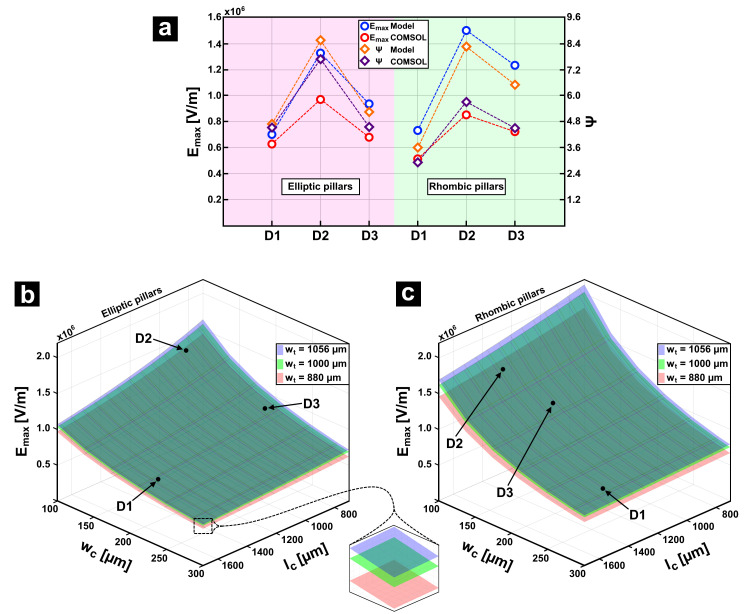
Evaluation of the DC-iEK designs described in [43] using the circuit model proposed in this work. (**a**) Maximum electric field magnitude and amplification factor for designs described in Table 3. Maximum electric field magnitude as function of $w_{c}$, $l_{c}$, and $w_{t}$ for designs with (**b**) elliptic pillars and (**c**) rhombic pillars. Labels D1, D2, and D3 indicate three different pillar array designs.

Figure 9a shows a comparison of the results for the electric field and amplification factor for all six designs described in Table 3 using both the circuit model and COMSOL simulations. The circuit model accurately predicts the trends for both pillar shapes and for all design variations. As Figure 9a only shows specific design cases, the resistance model was evaluated as a function of $w_{c}$ and $l_{c}$ for the three $w_{t}$ values presented in Table 3. Other parameters, such as channel length and applied voltage, were kept at 10.16 mm and 2500 V, respectively, as they were not subject to variation as presented in [43]. Figure 9b,c show surface plots for the results of this evaluation for elliptic and rhombic pillars, respectively. The three different colors for the surface plot represent the variation for the channel width: light red for $w_{t}$ = 880 µm, light green for $w_{t}$ = 1000 µm, and light blue for $w_{t}$ = 1056 µm. Black dots represent the position of the cases previously evaluated. However, here, a more general behavior is presented as the effect of each parameter is explicitly shown. It can be observed that both $w_{c}$ and $l_{c}$ have an impact on the magnitude of the electric field inside the device; however, it is evident that the variation of $w_{c}$ has a stronger influence. The presented zoom-in to Figure 9a gives a detail on the dependency with the channel width, $w_{t}$, and how the wider the channel, the higher the electric field, which is in accordance with the amplification factor as described by Equation (11).

These results are evidence of the model’s capacity to accurately predict the trends and general behavior of the electric field magnitude as a function of design parameters, for devices readily tested experimentally and reported in the literature. Furthermore, the main advantage of using the model proposed here is that there is no need for long (sometimes arbitrary or intuition-driven) numerical simulations, as analytical expressions can be readily evaluated and used to select the best design parameters based on the needs and resources of the user.

## 5. Conclusions

In this work, a model based on electric circuit theory was presented. It correctly predicted the electric field and voltage distribution trends throughout insulator-based electrokinetically driven microfluidic devices without the need for complex FEM-based simulations. While the electrical resistance equation served as the cornerstone of this work, the complete method yielded functions of voltage and electric field for the entire length of the microfluidic channel. Other relevant parameters such as current and amplification factor were also discussed. This model worked with a wide range of pillar array sizes and geometries, which were validated with FEM-based simulations built in COMSOL Multiphysics. Specifically, the predictive strength of this procedure was tested with eight cases designed for their expected behavior.

The model correctly predicted most relevant trends seen in the FEM-based simulations. Relevant correlations between electric field, current, electrical resistance, amplification factor, and insulating material were shown to be consistent between model and simulation. Moreover, some predicted values (e.g., amplification factor of case I) perfectly matched the prediction from the FEM-based simulation. Model-to-simulation deviations did occur on different degrees across cases. For the analysis of amplification factor, two-dimensional pillar arrays and single constriction structures showed single-digit percentual deviations (less than 6% error) at most. In contrast, channels with multiple, single-file constrictions deviated the most (e.g., amplification factor of case C, error less than 11%). This can be explained, as the latter designs present “caves” of conductive material that are unused by the current, which were assumed by the circuit model to be used by the current. It must be noted that although the model relies exclusively on the use of resistors (neglecting capacitive effects), easily correlating with DC-iEK and low-frequency AC-iEK scenarios, it can still provide estimates (albeit with reduced accuracy) of the electric field distribution in high-frequency AC-iEK cases. This is because both the electric conductivity and permittivity of insulating materials (e.g., PDMS, PMMA, glass, etc.) are much lower than those of suspending solutions used in iEK devices and so, current conduction always takes place chiefly in the solution.

Previous attempts at producing geometry-dependent analytical solutions for the electric field distribution along a cutline within insulator-based electrokinetic microdevices [34,35] relied on cumbersome symmetries. Moreover, such approaches produced closed-form solutions only for the most basic cases (i.e., a single gap produced by a column of two pillars) and could not be extrapolated to scenarios that included rectangular arrays of pillars. An additional limitation is that those solutions were only valid for circular pillars and triangular pillars. The method presented in this contribution is a general one which, by definition, can adapt to whatever pillar shape and be used in both single-gap and gap-array scenarios.

Further research will focus on refinement, distribution, and application of the circuit model. Refinement of the model will focus on the reduction and smoothing of the deviations just mentioned. Distribution of the circuit model can occur in the way of written work as this one was, or on the development of some human–machine interface that encapsulates the model into an easy-to-use program. Applied use of the circuit model can be, in turn, divided in two. Namely, the development of novel designs and further analysis of existing structures. For new designs, shorter iteration loops will unlock otherwise unreachable regions of the design possibility space. For already known arrangements, further analysis of trapping regions, voltage requirement predictions, and overall design performance will be performed with unprecedented efficiency.

Finally, although our efforts were directed towards simplifying the design and analysis process of insulator-based electrokinetically driven microfluidic devices, we produced a general method which can be used to approximate the electric field distribution along a cutline of whatever electrically stimulated device featuring one or several insulating structures on the current’s path.

## Figures and Tables

**Table 1:** Base equations of $f\left({x_{n}}^{\prime }\right)$ or $f\left({x_{n}}^{\prime \prime }\right)$ and relevant parameters to calculate $R_{g}$ of commonly used shapes.

Pillar Shape	$f\left({x_{n}}^{\prime }\right)$ $\mathrm{or}\;f\left({x_{n}}^{\prime \prime }\right)$	Range	Relevant Parameters
Rectangular	$\frac{w_{t}}{2a}-w_{1}$	$0\leq {x_{n}}^{\prime }\leq l_{p}$	Length $l_{p}$ Width $w_{1}$
Triangular (isosceles)	$\frac{w_{t}}{2a}-\frac{2w_{2}{x_{n}}^{\prime }}{l_{p}}$	$0\leq {x_{n}}^{\prime }\leq \frac{l_{p}}{2}$	Base $l_{p}$ Width $w_{2}$
	$\frac{w_{t}}{2a}-2w_{2}+\frac{2w_{2}{x_{n}}^{\prime }}{l_{p}}$	$\frac{l_{p}}{2}\leq {x_{n}}^{\prime }\leq l_{p}$	Base $l_{p}$ Width $w_{2}$
Circular	$\frac{w_{t}}{2a}-\sqrt{{\left(\frac{l_{p}}{2}\right)}^{2}-{\left({x_{n}}^{\prime \prime }\right)}^{2}}$	$\frac{-l_{p}}{2}\leq {x_{n}}^{\prime \prime }\leq \frac{l_{p}}{2}$	Radius $\frac{l_{p}}{2}=w_{3}$ Center position ${x_{n}}^{\prime \prime }=0$
Elliptical	$\frac{w_{t}}{2a}-\sqrt{{\left(w_{3}\right)}^{2}-{\left(\frac{2w_{3}}{l_{p}}\right)}^{2}{\left({x_{n}}^{\prime \prime }\right)}^{2}}$	$\frac{-l_{p}}{2}\leq {x_{n}}^{\prime \prime }\leq \frac{l_{p}}{2}$	Horizontal semi-major axis $\frac{l_{p}}{2}$ Vertical semi-major axis $w_{3}$ Center position ${x_{n}}^{\prime \prime }=0$

**Table 3:** Design parameters and equivalences used for assessing the applicability of the resistance model.

Pillar Shape	Variation	(a,b)	$w_{g}$ [µm]	$w_{2,3}$ [µm]	$l_{p}$ [µm]	$w_{t}$ [µm]	$w_{c}$ [µm]	$l_{c}$ [µm]
Elliptic	D1	(4, 16)	53.3	98.35	196.7	1000	213.2	3147.2
	D2	(4, 23)	25.6	97.2	72	880	102.4	1656
	D3	(12, 56)	16.9	35.55	28.2	1056	202.8	1579.2
Rhombic	D1	(4, 16)	68.8	90.6	181.2	1000	275.2	2899.2
	D2	(4, 23)	26.6	96.7	112.2	880	106.4	2580.6
	D3	(12, 56)	13.6	37.2	44	1056	163.2	2464

## Data Availability

Data is contained within the article or Supplementary Materials. Further inquiries can be directed to the corresponding author.

## Supplementary Materials

The following supporting information can be downloaded at: https://www.mdpi.com/article/10.3390/mi16111254/s1, Table S1: Relevant antiderivatives and definite integrals; Table S2: Resulting $R_{g}$ for commonly used shapes; Table S3: Resulting $R\left({x_{n}}^{\prime }\right)$ or $R\left({x_{n}}^{\prime \prime }\right)$ for commonly used shapes; Table S4: Specific numerical values and resulting electrical properties for the channels presented in Figure 5; Figure S1: Domain and boundary conditions used for the numerical modeling in COMSOL. Red and blue boundaries represent initial conditions.

## Abbreviations

EK	Electrokinetic
EO	Electroosmosis
EP	Electrophoresis
DEP	Dielectrophoresis
ER	Electrorotation
iEK	Insulator-based Electrokinetically Driven
FEM	Finite Element Method
DC	Direct Current
AC	Alternating Current
PDMS	Polydimethylsiloxane
