# Supplementary material for: On Using Electric Circuit Models to Analyze Electric Field Distributions in Insulator-Based Electrokinetically Driven Microfluidic Devices
Source: Micromachines (Basel). 2025 Nov 1;16(11):1254. doi: 10.3390/mi16111254 (PMC12654005; doi:10.3390/mi16111254)
Supplement: Supplementary file 1 [file micromachines-16-01254-s001.zip › micromachines-3924373-supplementary.pdf]

On Using Electric Circuit Models to Analyze Electric Field Distributions in Insulator-based Electrokinetically-driven Microfluidic Devices

J. Martin de-los-Santos-Ramirez[1], Ricardo Roberts[1], Vania G. Martinez-Gonzalez[1], Victor H. Perez-Gonzalez[1]*

[1]School of Engineering and Sciences, Tecnologico de Monterrey, Av. Eugenio Garza Sada 2501, Monterrey, N.L., 64700, México.

*Author to whom correspondence should be addressed.
Victor H. Perez-Gonzalez, vhpg@tec.mx

Supporting Information

Table S1. Relevant antiderivatives and definite integrals

Relevant for the rectangular pillar:

$$\int \frac{dx}{q} = \frac{x}{q} + C$$

$$\int_0^{l_p} \frac{dx}{q} = \frac{l_p}{q}$$

Relevant for the triangular pillar:

$$\int \frac{dx}{qx + h} = \frac{ln(qx + h)}{q} + C$$

$$\int_c^d \frac{dx}{qx + h} = \frac{ln(qd + h)}{q} - \frac{ln(qc + h)}{q}$$

Relevant for the circular and ellipsoidal pillars:

$$\int \frac{dx}{q - \sqrt{h^2 - (px)^2}} = \frac{-\sqrt{q^2 - h^2}\, tan^{-1}\left(\frac{px}{\sqrt{h^2 - (px)^2}}\right) + q\, tan^{-1}\left(\frac{qpx}{\sqrt{q^2 - h^2}\sqrt{h^2 - (px)^2}}\right) + q\, tan^{-1}\left(\frac{px}{\sqrt{q^2 - h^2}}\right)}{p\sqrt{q^2 - h^2}} + C$$

$$\int_{-\frac{l_p}{2}}^{\frac{l_p}{2}} \frac{dx}{q - \sqrt{h^2 - (px)^2}}$$

$$= \frac{-\sqrt{q^2 - h^2}\, tan^{-1}\left(\frac{p\frac{l_p}{2}}{\sqrt{h^2 - \left(p\frac{l_p}{2}\right)^2}}\right) + q\, tan^{-1}\left(\frac{qp\frac{l_p}{2}}{\sqrt{q^2 - h^2}\sqrt{h^2 - \left(p\frac{l_p}{2}\right)^2}}\right) + q\, tan^{-1}\left(\frac{p\frac{l_p}{2}}{\sqrt{q^2 - h^2}}\right)}{p\sqrt{q^2 - h^2}}$$

$$- \frac{-\sqrt{q^2 - h^2}\, tan^{-1}\left(\frac{p\frac{-l_p}{2}}{\sqrt{h^2 - \left(p\frac{-l_p}{2}\right)^2}}\right) + q\, tan^{-1}\left(\frac{qp\left(\frac{-l_p}{2}\right)}{\sqrt{q^2 - h^2}\sqrt{h^2 - \left(p\frac{-l_p}{2}\right)^2}}\right) + q\, tan^{-1}\left(\frac{p\frac{-l_p}{2}}{\sqrt{q^2 - h^2}}\right)}{p\sqrt{q^2 - h^2}}$$

Note that $tan^{-1}(0) = 0$. A simplified solution just doubles the integral that solves for the range $0 \le s \le \frac{l_p}{2}$. Therefore, we simplify as:

$$\int_{-\frac{l_p}{2}}^{\frac{l_p}{2}} \frac{dx}{q - \sqrt{h^2 - (px)^2}} = 2\left(\frac{-\sqrt{q^2 - h^2}\, tan^{-1}\left(\frac{p\frac{l_p}{2}}{\sqrt{h^2 - \left(p\frac{l_p}{2}\right)^2}} \right) + q\, tan^{-1}\left(\frac{qp\frac{l_p}{2}}{\sqrt{q^2 - h^2}\sqrt{h^2 - \left(p\frac{l_p}{2}\right)^2}} \right) + q\, tan^{-1}\left(\frac{p\frac{l_p}{2}}{\sqrt{q^2 - h^2}} \right)}{p\sqrt{q^2 - h^2}} \right)$$

For the particular case of the circle note that $p = 1$ and $h = \frac{l_p}{2}$, therefore:

$$\int_{-\frac{l_p}{2}}^{\frac{l_p}{2}} \frac{dx}{q - \sqrt{\left(\frac{l_p}{2}\right)^2 - x^2}} = 2\left(\frac{-\sqrt{q^2 - \left(\frac{l_p}{2}\right)^2}\, tan^{-1}\left(\frac{\frac{l_p}{2}}{\sqrt{\left(\frac{l_p}{2}\right)^2 - \left(\frac{l_p}{2}\right)^2}} \right) + q\, tan^{-1}\left(\frac{q\frac{l_p}{2}}{\sqrt{q^2 - \left(\frac{l_p}{2}\right)^2}\sqrt{\left(\frac{l_p}{2}\right)^2 - \left(\frac{l_p}{2}\right)^2}} \right) + q\, tan^{-1}\left(\frac{\frac{l_p}{2}}{\sqrt{q^2 - \left(\frac{l_p}{2}\right)^2}} \right)}{\sqrt{q^2 - \left(\frac{l_p}{2}\right)^2}} \right)$$

Which further simplifies into:

$$\int_{-\frac{l_p}{2}}^{\frac{l_p}{2}} \frac{dx}{q - \sqrt{\left(\frac{l_p}{2}\right)^2 - x^2}} = 2\left(\frac{-\sqrt{q^2 - \left(\frac{l_p}{2}\right)^2}\left(\frac{\pi}{2}\right) + q\left(\frac{\pi}{2}\right) + q\,tan^{-1}\left(\frac{l_p}{2\sqrt{q^2 - \left(\frac{l_p}{2}\right)^2}}\right)}{\sqrt{q^2 - \left(\frac{l_p}{2}\right)^2}}\right)$$

3

Table S2. Resulting R_g for commonly used shapes.

Pillar shape	$\boldsymbol{R_g}$
**Rectangular**	$\frac{1}{2\sigma D}\left(\frac{l_p}{\frac{w_t}{2a}-k_1}\right)$ Simplifying, $\frac{1}{\sigma D}\left(\frac{a l_p}{(w_t-2ak_1)}\right)$
**Triangular (isosceles)**	$\frac{1}{2\sigma D}\left(\frac{ln\left(-\frac{2k_2}{l_p}\frac{l_p}{2}+\frac{w_t}{2a}\right)}{-\frac{2k_2}{l_p}}-\frac{ln\left(\frac{w_t}{2a}\right)}{-\frac{2k_2}{l_p}}+\frac{ln\left(\frac{2k_2}{l_p}l_p+\frac{w_t}{2a}-2k_2\right)}{\frac{2k_2}{l_p}}-\frac{ln\left(\frac{2k_2}{l_p}\frac{l_p}{2}+\frac{w_t}{2a}-2k_2\right)}{\frac{2k_2}{l_p}}\right)$ Invoking symmetry, we simplify as: $\frac{1}{2\sigma D}\left(2\left(\frac{ln\left(-\frac{2k_2}{l_p}\frac{l_p}{2}+\frac{w_t}{2a}\right)}{-\frac{2k_2}{l_p}}-\frac{ln\left(\frac{w_t}{2a}\right)}{-\frac{2k_2}{l_p}}\right)\right)$ Further simplification $\frac{l_p}{2\sigma D k_2}\left(ln\left(\frac{w_t}{2a}\right)-ln\left(\frac{w_t}{2a}-k_2\right)\right)$

**Circular**	$\frac{1}{2\sigma D}\left(2\left(\frac{-\sqrt{\left(\frac{w_t}{2a}\right)^2-\left(\frac{l_p}{2}\right)^2}\left(\frac{\pi}{2}\right)+\left(\frac{w_t}{2a}\right)\left(\frac{\pi}{2}\right)+\left(\frac{w_t}{2a}\right)tan^{-1}\left(\frac{\frac{l_p}{2}}{\sqrt{\left(\frac{w_t}{2a}\right)^2-\left(\frac{l_p}{2}\right)^2}}\right)}{\sqrt{\left(\frac{w_t}{2a}\right)^2-\left(\frac{l_p}{2}\right)^2}}\right)\right)$
**Elliptical**	$\frac{1}{2\sigma D}\left(2\left(\frac{-\sqrt{\left(\frac{w_t}{2a}\right)^2-(k_3)^2}\,tan^{-1}\left(\frac{\left(\frac{4k_3}{l_p}\right)\left(\frac{l_p}{2}\right)}{\sqrt{(k_3)^2-\left(\frac{4k_3}{l_p}\frac{l_p}{2}\right)^2}}\right)+\left(\frac{w_t}{2a}\right)tan^{-1}\left(\frac{\left(\frac{w_t}{2a}\right)\left(\frac{4k_3}{l_p}\right)\left(\frac{l_p}{2}\right)}{\sqrt{\left(\frac{w_t}{2a}\right)^2-(k_3)^2}\sqrt{(k_3)^2-\left(\frac{4k_3}{l_p}\frac{l_p}{2}\right)^2}}\right)+\left(\frac{w_t}{2a}\right)tan^{-1}\left(\frac{\left(\frac{4k_3}{l_p}\right)\left(\frac{l_p}{2}\right)}{\sqrt{\left(\frac{w_t}{2a}\right)^2-(k_3)^2}}\right)}{\frac{4k_3}{l_p}\sqrt{\left(\frac{w_t}{2a}\right)^2-(k_3)^2}}\right)\right)$

9 **Table S3.** Resulting $R(x_n')$ or $R(x_n'')$ for commonly used shapes.

**Pillar shape**	$\boldsymbol{R(x_n')}$ **or** $\boldsymbol{R(x_n'')}$	
**Rectangle**	$\frac{x_n'}{2\sigma D\left(\frac{w_t}{2a}-k_1\right)}$ Simplifying, $\frac{1}{\sigma D}\left(\frac{a x_n'}{(w_t-2ak_1)}\right)$	
**Triangle**	$\frac{1}{2\sigma D}\left(\frac{ln\left(-\frac{2k_2}{l_p}x_n'+\frac{w_t}{2a}\right)}{-\frac{2k_2}{l_p}}-\frac{ln\left(\frac{w_t}{2a}\right)}{-\frac{2k_2}{l_p}}\right)$ Simplifying $\frac{l_p}{4\sigma D k_2}\left[ln\left(\frac{w_t}{2a}\right)-ln\left(\frac{w_t}{2a}-\frac{2k_2}{l_p}x_n'\right)\right]$	Range: $0 \le x_n' \le \frac{l_p}{2}$
	$\frac{ln\left(-\frac{2k_2}{l_p}\frac{l_p}{2}+\frac{w_t}{2a}\right)}{-\frac{2k_2}{l_p}}-\frac{ln\left(\frac{w_t}{2a}\right)}{-\frac{2k_2}{l_p}}+\frac{ln\left(\frac{2k_2}{l_p}x_n'+\frac{w_t}{2a}-2k_2\right)}{\frac{2k_2}{l_p}}-\frac{ln\left(\frac{2k_2}{l_p}\frac{l_p}{2}+\frac{w_t}{2a}-2k_2\right)}{\frac{2k_2}{l_p}}$ Simplifying $\frac{l_p}{4\sigma D k_2}\left[ln\left(\frac{w_t}{2a}\right)-2ln\left(\frac{w_t}{2a}-k_2\right)+ln\left(\frac{w_t}{2a}+\frac{2k_2}{l_p}x_n'-2k_2\right)\right]$	Range: $\frac{l_p}{2} \le x_n' \le l_p$

Circle

$$\frac{1}{2\sigma D}\left(\frac{-\sqrt{\left(\frac{w_t}{2a}\right)^2-\left(\frac{l_p}{2}\right)^2}\,tan^{-1}\left(\frac{x_n''}{\sqrt{\left(\frac{l_p}{2}\right)^2-(x_n'')^2}}\right)+\left(\frac{w_t}{2a}\right)tan^{-1}\left(\frac{\left(\frac{w_t}{2a}\right)x_n''}{\sqrt{\left(\frac{w_t}{2a}\right)^2-\left(\frac{l_p}{2}\right)^2}\sqrt{\left(\frac{l_p}{2}\right)^2-(x_n'')^2}}\right)+\left(\frac{w_t}{2a}\right)tan^{-1}\left(\frac{x_n''}{\sqrt{\left(\frac{w_t}{2a}\right)^2-\left(\frac{l_p}{2}\right)^2}}\right)}{\sqrt{\left(\frac{w_t}{2a}\right)^2-\left(\frac{l_p}{2}\right)^2}}\right)$$

$$-\frac{1}{2\sigma D}\left(\frac{-\sqrt{\left(\frac{w_t}{2a}\right)^2-\left(\frac{l_p}{2}\right)^2}\,tan^{-1}\left(\frac{\frac{-l_p}{2}}{\sqrt{\left(\frac{l_p}{2}\right)^2-\left(\frac{-l_p}{2}\right)^2}}\right)+\left(\frac{w_t}{2a}\right)tan^{-1}\left(\frac{\left(\frac{w_t}{2a}\right)\left(\frac{-l_p}{2}\right)}{\sqrt{\left(\frac{w_t}{2a}\right)^2-\left(\frac{l_p}{2}\right)^2}\sqrt{\left(\frac{l_p}{2}\right)^2-\left(\frac{-l_p}{2}\right)^2}}\right)+\left(\frac{w_t}{2a}\right)tan^{-1}\left(\frac{\frac{-l_p}{2}}{\sqrt{\left(\frac{w_t}{2a}\right)^2-\left(\frac{l_p}{2}\right)^2}}\right)}{\sqrt{\left(\frac{w_b}{2a}\right)^2-\left(\frac{l_p}{2}\right)^2}}\right)$$

Simplifying,

$$\frac{1}{2\sigma D}\left(\frac{-\sqrt{\left(\frac{w_t}{2a}\right)^2-\left(\frac{l_p}{2}\right)^2}\,tan^{-1}\left(\frac{x_n''}{\sqrt{\left(\frac{l_p}{2}\right)^2-(x_n'')^2}}\right)+\left(\frac{w_t}{2a}\right)tan^{-1}\left(\frac{\left(\frac{w_t}{2a}\right)x_n''}{\sqrt{\left(\frac{w_t}{2a}\right)^2-\left(\frac{l_p}{2}\right)^2}\sqrt{\left(\frac{l_p}{2}\right)^2-(x_n'')^2}}\right)+\left(\frac{w_t}{2a}\right)tan^{-1}\left(\frac{x_n''}{\sqrt{\left(\frac{w_t}{2a}\right)^2-\left(\frac{l_p}{2}\right)^2}}\right)}{\sqrt{\left(\frac{w_t}{2a}\right)^2-\left(\frac{l_p}{2}\right)^2}}\right)$$

	$-\frac{1}{2\sigma D}\left(\frac{-\sqrt{\left(\frac{w_t}{2a}\right)^2-\left(\frac{l_p}{2}\right)^2}\left(\frac{-\pi}{2}\right)+\left(\frac{w_t}{2a}\right)\left(\frac{-\pi}{2}\right)+\left(\frac{w_t}{2a}\right)tan^{-1}\left(\frac{\frac{-l_p}{2}}{\sqrt{\left(\frac{w_t}{2a}\right)^2-\left(\frac{l_p}{2}\right)^2}}\right)}{\sqrt{\left(\frac{w_t}{2a}\right)^2-\left(\frac{l_p}{2}\right)^2}}\right)$
**Ellipse**	$\frac{1}{2\sigma D}\left(\frac{-\sqrt{\left(\frac{w_t}{2a}\right)^2-(k_3)^2}\,tan^{-1}\left(\frac{\left(\frac{2k_3}{l_p}\right)x_n''}{\sqrt{((k_3))^2-\left(\left(\frac{2k_3}{l_p}\right)x_n''\right)^2}}\right)+\left(\frac{w_t}{2a}\right)tan^{-1}\left(\frac{\left(\frac{w_t}{2a}\right)\left(\frac{2k_3}{l_p}\right)x_n''}{\sqrt{\left(\frac{w_t}{2a}\right)^2-(k_3)^2}\sqrt{(k_3)^2-\left(\left(\frac{2k_3}{l_p}\right)x_n''\right)^2}}\right)+\left(\frac{w_t}{2a}\right)tan^{-1}\left(\frac{\left(\frac{2k_3}{l_p}\right)x_n''}{\sqrt{\left(\frac{w_t}{2a}\right)^2-(k_3)^2}}\right)}{\left(\frac{2k_3}{l_p}\right)\sqrt{\left(\frac{w_t}{2a}\right)^2-(k_3)^2}} - \frac{-\sqrt{\left(\frac{w_t}{2a}\right)^2-(k_3)^2}\,tan^{-1}\left(\frac{\left(\frac{2k_3}{l_p}\right)\left(\frac{-l_p}{2}\right)}{\sqrt{(k_3)^2-\left(\left(\frac{2k_3}{l_p}\right)\left(\frac{-l_p}{2}\right)\right)^2}}\right)+\left(\frac{w_t}{2a}\right)tan^{-1}\left(\frac{\left(\frac{w_t}{2a}\right)\left(\frac{2k_3}{l_p}\right)\left(\frac{-l_p}{2}\right)}{\sqrt{\left(\frac{w_t}{2a}\right)^2-(k_3)^2}\sqrt{(k_3)^2-\left(\left(\frac{2k_3}{l_p}\right)\left(\frac{-l_p}{2}\right)\right)^2}}\right)+\left(\frac{w_t}{2a}\right)tan^{-1}\left(\frac{\left(\frac{2k_3}{l_p}\right)\left(\frac{-l_p}{2}\right)}{\sqrt{\left(\frac{w_t}{2a}\right)^2-(k_3)^2}}\right)}{\left(\frac{2k_3}{l_p}\right)\sqrt{\left(\frac{w_t}{2a}\right)^2-(k_3)^2}}\right)$

Table S4. Specific numerical values and resulting electrical properties for the channels presented in Figure 5. Underlined values show the cases with the minimum disparity between model and simulation. Common dimensional parameters for all cases are: $w_t = 1$ mm, $l_l = l_r = 4.6$ mm, $l_c = 800$ μm, $D = 20$ μm, and $\sigma = 100$ μS/cm.

Channel Design	Array Size (a,b)	Relevant Dimensional Parameters [μm]	PDMS (%)	R_T [MΩ]	Ψ	E_{max} [V/cm]
A	(5,4)	$l_p = 150$<br>$w_1 = 75$<br>$w_g = 50$	4.500	Circuit: 59.00<br>COMSOL:60.87	Circuit: 4.00<br>COMSOL: 3.92	Circuit: 339<br>COMSOL: 323
B	(5,3)	$l_p = 150$<br>$w_1 = 75$<br>$w_g = 50$	3.375	Circuit: 56.75<br>COMSOL:58.20	Circuit: 4.00<br>COMSOL: 3.91	Circuit: 352<br>COMSOL: 337
C	(1,4)	$l_p = 150$<br>$w_1 = 375$<br>$w_g = 250$	4.500	Circuit: 59.00<br>COMSOL:64.22	Circuit: 4.00<br>COMSOL: 4.47	Circuit: 339<br>COMSOL: 323
D	(1,1)	$l_p = 600$<br>$w_1 = 375$<br>$w_g = 250$	4.500	Circuit: 59.00<br>COMSOL: 62.17	Circuit: 4.00<br>COMSOL: 3.99	Circuit: 339<br>COMSOL: 321
E	(1,1)	$l_p = 800$<br>$w_1 = 281.25$<br>$w_g = 437.5$	4.500	Circuit: 55.14<br>COMSOL:56.71	Circuit: 2.29<br>COMSOL: 2.28	Circuit: 207<br>COMSOL: 201
F	(1,1)	$l_p = 800$<br>$w_1 = 346.155$<br>$w_g = 307.69$	5.538	Circuit: 59.00<br>COMSOL:61.57	Circuit: 3.25<br>COMSOL: 3.25	Circuit: 275<br>COMSOL: 264
G	(1,1)	$l_p = 800$<br>$w_1 = 375$<br>$w_g = 250$	6.000	Circuit: 62.00<br>COMSOL:65.17	Circuit: 4.00<br>COMSOL: 3.99	Circuit: 322<br>COMSOL: 306
H	(1,1)	$l_p = 800$<br>$w_1 = 383.15$<br>$w_g = 233.70$	6.130	Circuit: 63.12<br>COMSOL: 66.50	Circuit: 4.26<br>COMSOL: 4.25	Circuit: 339<br>COMSOL: 320
I	(1,1)	$l_p = 800$<br>$w_1 = 389.27$<br>$w_g = 221.46$	6.228	Circuit: 64.06<br>COMSOL: 67.61	Circuit: 4.51<br>COMSOL: 4.51	Circuit: 352<br>COMSOL: 333

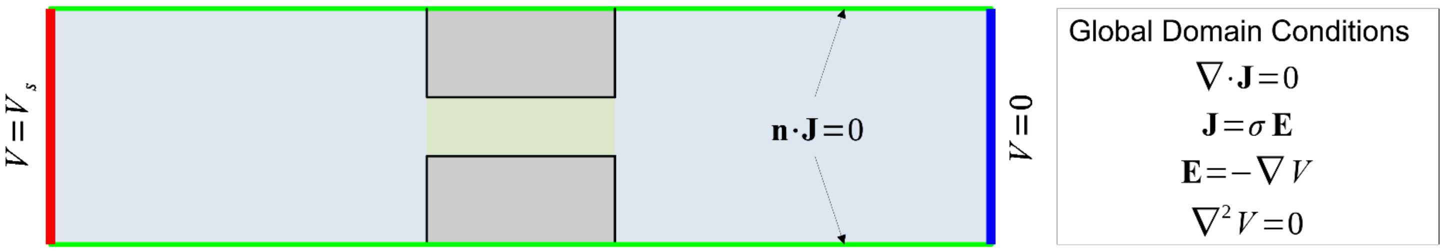

Figure S1. Domain and boundary conditions used for the numerical COMSOL model. The red boundary represents electric potential, blue boundary is ground, and green boundary represents electrical insulation. Two posts are used herein for illustration purposes only. Post arrays were constructed as detailed in Section 4.1 and Table S4. In the equations, $\mathbf{J}$ represents current density, ρ_v is volume charge density, σ is electrical conductivity, $\mathbf{E}$ is electric field intensity, $\mathbf{n}$ is the outward normal unit vector, and V represents the electric potential.
